# Medical and Physician Assistant Student Competence in Basic Life Support: Opportunities to Improve Cardiopulmonary Resuscitation Training

**DOI:** 10.5811/westjem.2020.11.48536

**Published:** 2020-12-15

**Authors:** Rohit Gupta, Stephanie DeSandro, Neil A. Doherty, Aimee K. Gardner, M. Tyson Pillow

**Affiliations:** * Baylor College of Medicine, School of Medicine, Houston, Texas; † Baylor College of Medicine, Department of Emergency Medicine, Houston, Texas

## Abstract

**Introduction:**

Medical and physician assistant (PA) students are often required to have Basic Life Support (BLS) education prior to engaging in patient care. Given the potential role of students in resuscitations, it is imperative to ensure that current BLS training prepares students to provide effective cardiopulmonary resuscitation (CPR). The objective of this study was to assess whether current BLS training produces student providers who can deliver BLS in an American Heart Association (AHA) guideline-adherent manner.

**Methods:**

Students at a US medical school were recruited by convenience sampling. BLS performance immediately following a standard AHA BLS training course was evaluated during a two-minute CPR cycle using manikins. We also collected information on demographics, previous BLS training attendance, perceived comfort in providing CPR, and prior experiences in healthcare and providing or observing CPR.

**Results:**

Among 80 participants, we found that compression rate, depth, and inter-compression recoil were AHA guideline-adherent for 90.0%, 68.8%, and 79.3% of total compression time, respectively. Mean hands-off time was also within AHA guidelines. Mean number of unsuccessful ventilations per cycle was 2.2. Additionally, 44.3% of ventilations delivered were of adequate tidal volume, 12.2% were excessive, and 41.0% were inadequate. Past BLS course attendance, prior healthcare certification, and previous provision of real-life CPR were associated with improved performance.

**Conclusion:**

Following BLS training, medical and PA students met a majority of AHA compressions guidelines, but not ventilations guidelines, for over 70% of CPR cycles. Maintaining compression depth and providing appropriate ventilation volumes represent areas of improvement. Conducting regular practice and involving students in real-life CPR may improve performance.

## INTRODUCTION

Each year in the United States, approximately 209,000 patients experience a cardiac arrest in the hospital and undergo subsequent resuscitative efforts. Another 360,000 will arrest out of the hospital setting and rely on individuals in the community to recognize the emergency and initiate the appropriate interventions.^1^ Provision of Basic Life Support (BLS) consisting of rapid, deep, chest compressions with appropriate ventilation is the cornerstone of resuscitation in both hospital and community settings. Based on outcome analyses, clear guidelines to administer BLS have been developed. The American Heart Association (AHA) recommends that adult cardiopulmonary resuscitation (CPR) be performed with compressions at a depth of at least two inches and at a rate of at least 100 per minute, allowing for full recoil of the patient’s chest.^2^,^3^ Additionally, ventilation of the lungs should be provided over 1–2 seconds and should not exceed the level of maximal chest rise.^4^,^5^

Although advanced interventions such as endotracheal intubation and pharmacologic therapies are often provided by higher level providers during resuscitation efforts, multiple prospective and retrospective analyses have demonstrated that the provision of BLS techniques remains the outcome-determining factor for patients experiencing cardiac arrest.^6^–^8^ This includes initiation of effective chest compressions in the first minutes of a cardiac arrest.^8^–^10^ Therefore, deficits in the BLS skillset of health providers can have a major impact on healthcare institutions and the communities they serve by not only depriving cardiac arrest victims of the benefits of treatment, but also potentially increasing mortality.

Two such groups of health providers that must have BLS competency are medical students and physician assistant (PA) students. The Association of American Medical Colleges (AAMC) identifies the ability to recognize the need for emergent care and initiate early management in decompensating patients as required skills that medical school graduates must be able to perform on the first day of residency.^11^ The AAMC further specifies that the ability to provide basic and advanced life support is derived directly from these expected areas of competency. The Physician Assistant Education Association Presidents Commission has identified similar requirements for PA graduates.^12^ Given that medical and PA students are essential members highly involved in care teams and may be involved in resuscitations, it is imperative to ensure that these individuals meet the high standards for effective BLS administration as well as maintain BLS knowledge throughout their tenure treating patients. This is also important to ensure faculty are confident when entrusting responsibility to these students during resuscitations.

However, the extent to which BLS standards and expectations are being met following current health professions BLS training is largely unknown. Determining this as well as other factors that contribute to BLS competence and long-term retention may not only improve delivery of CPR by medical students, but also instill trust in faculty in involving students in resuscitations. Here, we assessed the adequacy of current BLS training in health professions curricula by measuring CPR performance metrics in medical and PA students following completion of a standard AHA BLS course. We also explored whether previous CPR experience impacts performance, and whether significant differences exist between PA and medical student performance.

**Population Health Research Capsule**

What do we already know about this issue?

Medical and physician’s assistant (PA) students are important members of patient care teams who may be involved in resuscitations during clinical rotations.

What was the research question?

Does current Basic Life Support (BLS) training produce competency in medical and PA students, and what are areas for improvement?

What was the major finding of the study?

Students were deficient in areas of BLS, and previous BLS experience was associated with better performance.

How does this improve population health?

Current BLS education in medical curriculum may need refinement for clinicians to be confident in involving medical and PA students in resuscitations.

## METHODS

### Participants and Study Setting

This study was conducted at a medical and health professions school located in the United States. A convenience sample of all graduating fourth-year medical students and all first-year PA students at the school (a total of 184 and 40 individuals, respectively, eligible to participate) were invited to participate in the study at the conclusion of their requisite, fully guideline-compliant AHA BLS training classes offered as part of their curricula in March 2015. A total of eight classes across one week were taught, with both PA and medical students mixed in each class. While the specific class instructors varied occasionally across the classes, all courses were taught by AHA-certified BLS instructors. Furthermore, class instruction materials were standardized across all classes, which included lecture-based BLS instruction via AHA videos covering all aspects of BLS (eg, scene assessment, compressions, ventilations, automated external defibrillator use, etc.) as well as standardized times for hands-on practice with manikins. Participation in the study was voluntary; no incentive was provided, and participation had no effect on academic or professional standing.

### Assessment of BLS Competency

**Figure 1:**
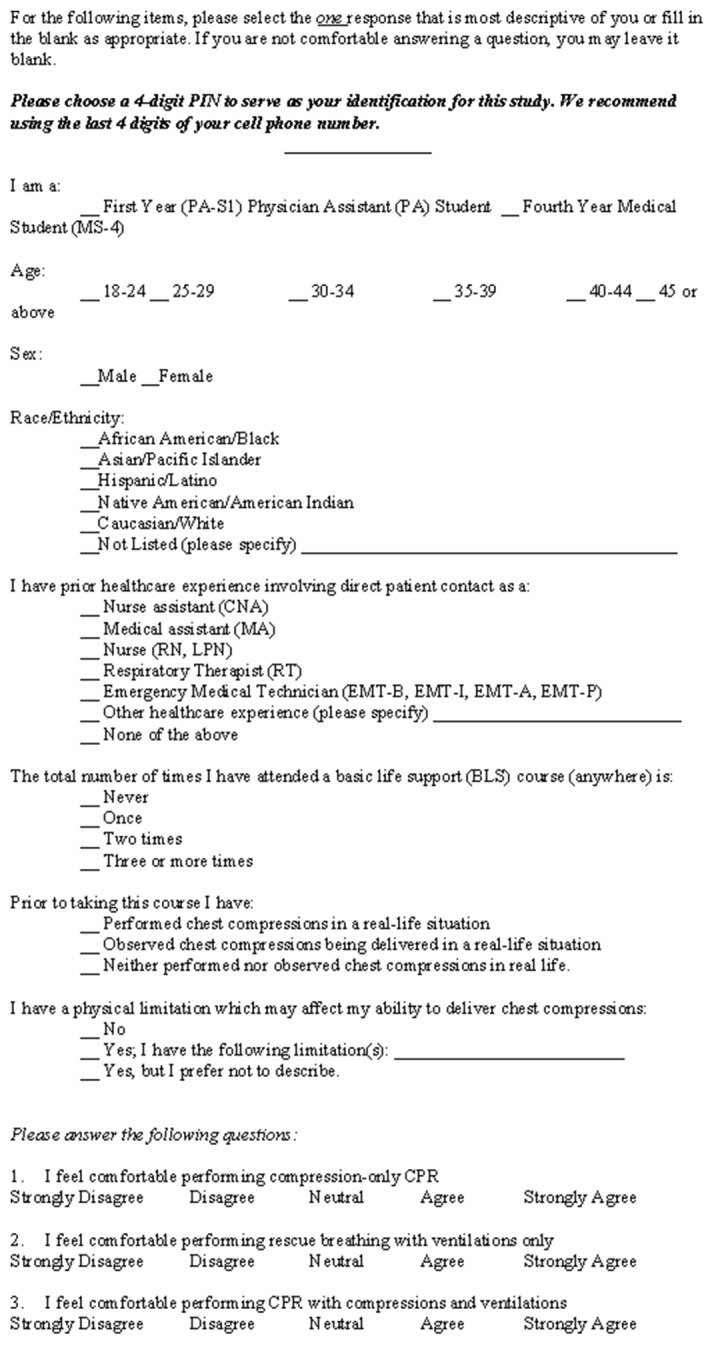
Pre-assessment Basic Life Support survey of medical students and physician assistant students.

Within one hour of completing the BLS training course, participants completed a pre-assessment survey requesting demographic information, previous BLS class attendance, previously received healthcare certifications (eg, emergency medical technician), perceived comfort in performing each component of CPR, and prior experiences providing or observing CPR (Figure 1). Participants were then instructed to complete a two-minute cycle of monitored adult CPR using bag-valve mask (BVM) for ventilation. We used two ResusciAnne QCPR manikins (Laerdal Medical, Stavanger, Norway) to obtain objective data regarding the parameters of compressions and ventilations delivered including chest compression, hand placement, depth, rate, presence of appropriate recoil, total CPR hands-off time, ventilation success, and ventilatory tidal volume. Following this assessment, participants were given the opportunity to see their individual results and receive feedback from trained AHA instructors observing their performance, if they desired.

### Statistical Analysis

Parametric data interpreted on the QCPR manikin SimPad was exported to statistical software for data analysis and are reported with standard deviation. In addition to comparing student CPR performance to published AHA BLS standards, we examined relationships between performance and student gender, prior BLS classes, prior healthcare certifications, prior observations of CPR, and prior experience with CPR. Descriptive statistics, independent t-tests, and one-way analysis of variance (ANOVA) were performed with SPSS version 24.0 (IBM Corporation, Armonk, NY).

## RESULTS

A total of 48 fourth-year medical students and 32 first-year PA students participated in the study. The sample demographics and prior healthcare certifications are listed in Table 1. The majority of students (75%) indicated they had attended a BLS course two or more times. When asked about prior BLS experiences in real-life situations, 60% of students reported observing compressions, and 37.5% indicated they had administered compressions themselves. The majority of students agreed or strongly agreed that that they felt comfortable performing compressions-based CPR (88%), providing rescue breaths with ventilation (78.8%), and performing CPR with both compressions and ventilations (78.8%).

Outcomes of the two-minute CPR cycle are detailed in Table 2 (compression metrics) and Table 3 (ventilation metrics), along with the corresponding AHA guidelines for each metric measured.

Statistically significant relationships were noted between CPR characteristics and student gender, prior healthcare certifications, attendance of prior BLS classes, and prior CPR experience. Overall, male gender was associated with significantly greater mean compression depth compared to female gender (56.5 millimeters [mm] ±6.7 vs 50.3 mm ±6.5; *P* < 0.001) as well as maintenance of appropriate depth during the entire two-minute cycle (82.3% ±34.6 of male cycles vs 57.3% ±36.2 of female cycles; *P* < 0.01). After further stratifying these data by student type (medical vs PA student), we found that the significant differences in compression metrics between genders was maintained for medical students, but no differences were observed between male and female PA students. No significant differences emerged among the other metrics based on gender.

Students with prior healthcare certifications (Table 1), compared to those without prior certifications, had significantly higher mean compression rates per minute (149.4 ±20.0 vs 126.5 ±18.6; *P* < 0.001), less hands-off time (8.4 ±1.4 seconds vs 9.8 ±1.9 seconds; *P* < 0.01), higher frequency of correct hand position (90.7% ±25.8 vs 73.3% ±37.1; *P* < 0.05), higher number of CPR cycles completed in five minutes (5.4 ±0.8 vs 4.4 ±0.7; *P* < 0.001), and higher ventilation mean rate per minute (4.3 ±1.6 vs 3.2 ±1.6; *P* < 0.01).

Similarly, the number of BLS courses attended in the past was correlated with improved performance on a number of parameters, including compression mean rate (*P* < 0.001), correct hand positioning (*P* < 0.05), ventilation mean volume (*P* < 0.01), and missed ventilations (*P* < 0.05). Subgroup analysis revealed that the positive overall effects of prior BLS attendance were primarily a result of differences seen within the PA student cohort. Furthermore, compression mean rate (*P* < 0.05), compression mean depth (*P* < 0.05), maintenance of appropriate depth (*P* < 0.01), total CPR cycles (*P* < 0.01) and number of ventilations given (*P* < 0.05) all were significantly positively correlated with number of prior BLS classes among PA students.

Previous observation of CPR did not correlate with performance; however, provision of real-life CPR did. Specifically, participation in CPR was associated with significant increases in mean compression depth (56.4 mm ±6.0 vs 51.2 mm ±7.3; *P* < 0.001) and maintenance of appropriate depth during the entire two-minute cycle (86.1% ±28.6 vs 58.4% ±38.4; *P* < 0.001). No significant differences emerged in the other measures based on CPR participation or observation.

We also compared the performances of medical students and PA students, summarized in Table 2 and Table 3. Medical students were found to compress at a greater mean depth than PA students, whereas PA students compressed at a greater mean rate per minute. PA students exhibited a significantly higher average ventilation rate per two-minute cycle, less hands-off time, higher number of completed CPR cycles, and higher rate of correct hand positioning when compared to medical students. No significant differences between student types were observed in the other parameters measured.

## DISCUSSION

Our goal in this study was to investigate the adequacy of CPR training in health professions curricula. We assessed this by measuring the BLS proficiency of medical and PA students immediately following a standard AHA BLS training course. To our knowledge, this is the first study that looks at competency-based skills attainment for BLS in either medical students or PA students immediately following standard BLS training. The results of this study suggest that, despite significant differences in performance found between student types, the current AHA CPR training produces medical and PA students who each are adequate providers of compressions, defined as meeting a majority of AHA guidelines in at least 70% of compression cycles. However, the training did not produce adequate providers of ventilations by this definition. The assessment of specific CPR metrics identified many areas of weakness in performance, indicating students are imperfect when delivering CPR. Therefore, BLS classes for medical and PA students can be improved in order to remedy these deficiencies. We found that previous attendance of BLS courses, having a prior healthcare certification, and prior experience providing compressions on a patient, but not observing compressions, were associated with significantly higher BLS performance. Taken together, our study suggests that AHA BLS courses prepare medical and PA students to adequately deliver quality compressions, but there are opportunities for overall improvement both via training and experiential learning.

Overall, compression rate was satisfactory, with 90% of students maintaining a guideline-adherent rate for the duration of the two-minute cycle. However, despite mean depth of compressions and variations in depth above recommendations, student performance was lacking in maintaining an appropriate compression depth throughout CPR performance. Similarly, inadequate ventilation volume and missed ventilations were common. It is well established that high-quality compressions significantly impact patient outcomes in CPR.^13^ In addition, the ability to ventilate a patient is a critical skill for acute patient deterioration situations, especially when other ancillary staff are unavailable. Thus, focused curriculum reform may be necessary for BLS training programs. It is difficult to assess the reasons why students lacked in performance on certain compression and ventilation measures; however, one potential remedy may be increasing the amount of hands-on time students have with the manikins during BLS training. In addition, studies have demonstrated that using CPR feedback/prompt devices or similar teacher-to-student CPR feedback during hands-on training improves performance.^14^,^15^ Implementing these strategies during BLS training may, therefore, improve performance among medical and PA students.

Another possibility to improve the current BLS training model may be implementing dyad training, which entails cooperative learning in pairs. Wang et al demonstrated the utility of this model in Advanced Cardiac Life Support (ACLS) training of medical students.^16^ Using a dyadic training model during ACLS training resulted in significantly improved resuscitation scores (an overall measure of teamwork), resuscitation skills, and leadership. However, Wang et al did not report specifically whether this method improved exact compression and ventilation parameters. For BLS training, implementing a dyadic model may involve splitting students into pairs and alternating between each individual on simulated practice, followed by shared discussion. Further studies are warranted to explore whether such a change in training design can improve medical and PA student BLS performance.

Training of medical and PA students in BLS may also benefit from distributed practice of CPR. Lin et al studied the effects of such practice among pediatric healthcare providers.^15^ Practicing CPR for two minutes on manikins at least once per month, in combination with real-time feedback, resulted in significantly improved adult and infant CPR quality across a vast majority of CPR parameters compared to those who did not practice CPR. This improved performance lasted throughout the 12-month study period. Similarly, Nishiyama et al found that a 15-minute refresher BLS training of medical students resulted in significant improvement in certain compression proficiency measurements, up to one year after the refresher.^17^ This is consistent with the findings of our study that prior BLS course attendance was significantly associated with improved CPR performance. Overall, this suggests that, although medical and PA students are imperfect CPR providers immediately after BLS training, their skills may improve with deliberate, frequent practice. Such practice may be implemented into the normal clinical instruction of these students to ensure regularity and adherence.

Another potential source of active BLS skills practice for medical and PA students may be actively involving students during patient resuscitations they encounter naturally in their clinical duties. Our study found that a majority of students had observed compressions being delivered to a patient, but only 37.5% had administered compressions themselves. Furthermore, experience delivering compressions on a patient in the past as well as having a prior healthcare certification significantly correlated with higher performance in multiple measures of compression adequacy; however, prior experience only observing compressions did not. Together, this suggests that involving students in real-life hands-on experiences, such as actual resuscitations, improves their CPR performance over time, but a majority of students do not get this opportunity.

Unfortunately, given that this study demonstrated that student performance lacks in multiple CPR parameters immediately after BLS training, simply allowing students to perform CPR on arrested patients without ensuring BLS excellence may risk patient outcomes. Furthermore, even if students were allowed to administer CPR, in a previous study our group found that >35% of fourth-year medical students were reluctant to participate in resuscitations, as they felt unprepared. Training programs would be wise to adopt competency-based assessments along with regular practice to ensure proficiency and preparation in these areas rather than relying on certifications after training courses. This is especially salient given we found that a vast majority of students in our study self-reported confidence in their CPR skills.

Finally, we did not explore how BLS skills among medical and PA students change over time after the immediate period following BLS training. Previous studies have demonstrated that after training, self-reported confidence in BLS skills and proficiency in BLS skills decline.^1^ As discussed above, regular practice may be necessary for students to not only retain BLS skills, but also improve upon them. Longitudinal investigations of multiple cohorts of medical and PA students in a controlled, experimental setting are needed to help elucidate changes in BLS skills over time and how skill decay can be counteracted.

## LIMITATIONS

Although this study reports granular data on the ability of first-year PA students and fourth-year medical students to perform CPR-related skills following BLS training, there are a number of limitations. First, these data represent only a snapshot of performance in a simulated setting. It is unknown how students would perform in each of these areas in the face of a true clinical scenario. Along similar lines, some useful metrics, such as chest rise and fall and patient-centered outcomes, were not measured in the study. The baseline BLS skill competence of students was not measured prior to the BLS course, so it is difficult to assess whether students significantly improved in skills after taking the course. However, this is a minor limitation, as the goal of the study was to evaluate whether students are competent up to BLS guidelines following AHA BLS training in the health curricula, not whether there is a significant improvement from baseline. The impact of feedback and reassessment on student performance was not measured, either. Additionally, this study was conducted at a single US medical/health professions school; complicating this, selection bias may have skewed the data, as these results capture data from a convenience sample of trainees who volunteered to be included after participating in a standardized AHA BLS course.

## CONCLUSION

The current AHA BLS training provided to fourth-year medical students and first-year PA students produces providers who are capable of delivering compressions in an AHA guideline-adherent manner for the majority of a two-minute CPR cycle. However, inadequate performance in certain compression and many ventilation measurements demonstrate areas of improvement for BLS training programs. Previous attendance of BLS trainings, prior healthcare certification, and prior real-life experience in administering CPR were found to be predictive of student BLS competency, but previous experience simply observing CPR being performed was not.

## Figures and Tables

**Table 1:** Participant demographic information.

Characteristics	Medical students, N (%)	Physician assistant students, N (%)	P-value
Age			<0.01
18–24	2 (4.2%)	13 (40.6%)	
25–29	40 (83.3%)	14 (43.8%)	
30–34	6 (12.5%)	3 (9.4%)	
35–39	0 (0%)	1 (3.1%)	
40–44	0 (0%)	1 (3.1%)	
Gender			<0.01
Female	20 (41.7%)	25 (78.1%)	
Male	28 (58.3%)	7 (21.9%)	
Race/ethnicity			0.76
Black	2 (4.2%)	1 (3.1%)	
Asian/Pacific Islander	12 (25.0%)	6 (12.5%)	
Hispanic/Latino	5 (10.4%)	5 (10.4%)	
Native American	1 (2.1%)	0 (0%)	
White	24 (50%)	19 (39.6%)	
Other	4 (8.3%)	1 (2.1%)	
Prior healthcare certification			<0.01
Clinical Nurse Assistant	0 (0%)	10 (31.3%)	
Medical Assistant	1 (2.1%)	2 (6.3%)	
Registered Nurse	0 (0%)	1 (3.1%)	
Emergency Medical Technician	4 (8.3%)	6 (18.8%)	
Other	2 (4.2%)	7 (21.9%)	
None	41 (85.4%)	6 (18.8%)	

**Table 2:** Compression data across student type.

	All students (N=80)	Medical students (N=48)	Physician assistant students (N=32)	AHA guidelines
Mean compression rate (#/minute)	134.5 ± 21.9	**121.1 ± 11.7**	154.6 ± 17.9**	> 100/minute
Appropriate rate (%/cycle)	90.0 ± 30.2	88.0 ± 33.4	94.0 ± 24.6	100%
Mean compression depth (mm)	53.1 ± 7.2	**54.7 ± 6.8**	50.8 ± 7.4*	> 50.8 mm
Appropriate depth (%/cycle)	68.8 ± 37.4	74.8 ± 34.9	59.8 ± 39.6	100%
Appropriate recoil (%/cycle)	79.3 ± 24.4	78.0 ± 26.4	81.2 ± 21.4	100%
Hands-off time (sec)	9.3 ± 1.8	**10.0 ± 1.8**	8.3 ± 1.3**	< 10 seconds
Correct hand position (%/cycle)	79.4 ± 34.4	**67.9 ± 38.4**	96.6 ± 16.6**	100%
CPR cycles completed (#/cycle)	4.8 ± 0.9	**4.2 ± 0.6**	5.5 ± 0.6**	5

Note: Bold = comparisons between medical student and physician assistant students are significant at levels:

* P < 0.05;

** P < 0.001.

*AHA*, American Heart Association; *CPR*, cardiopulmonary resuscitation; *mm*, millimeter.

**Table 3:** Ventilation data across student type.

	All students (N=80)	Medical students (N=48)	Physician assistant students (N=32)	AHA guidelines
Ventilation mean volume (mL)	458.5 ± 161.9	451.8 ± 176.0	468.5 ± 142.2	450 – 550 mL
Excessive ventilation (%/cycle)	12.2 ± 22.1	12.1 ± 22.9	12.4 ± 21.1	0%
Appropriateness of ventilation (%/cycle)	44.3 ± 31.9	40.9 ± 34.0	49.3 ± 28.1	100%
Inadequate ventilation (%/cycle)	41.0 ± 35.2	42.8 ± 38.5	38.3 ± 30.0	0%
Ventilation mean rate (#/min)	3.5 ± 1.7	**3.0 ± 1.5**	4.4 ± 1.5**	5/min
Missed Ventilations	2.2 ± 2.4	2.4 ± 2.5	1.8 ± 2.2	0

Note: Bold = comparisons between medical student and physician assistant students are significant at levels:

* P < 0.05;

** P < 0.001.

*AHA*, American Heart Association; *mL*, milliliter; *min*, minute.
